# Paeonol inhibits the development of oral squamous cell carcinoma through the PI3K/AKT signaling pathway

**DOI:** 10.3389/fcell.2026.1747424

**Published:** 2026-03-04

**Authors:** Changyue Liu, Xuelin Mou, Jiaming Liu, Yuehan Wu, Jinpeng Han, Ren Li, Yuxia Gao, Ying Liu

**Affiliations:** 1 Department of Stomatology,Affiliated Hospital of North Sichuan Medical College, Nanchong, China; 2 Department of Stomatology,North Sichuan Medical College, Nanchong, China; 3 Jiangxi Provincial Key Laboratory of Oral Diseases, Department of Stomatology, The First Affiliated Hospital, Jiangxi Medical College, Nanchang University, Nanchang, China; 4 Department of Stomatology,Changsha Medical University, Changsha, China

**Keywords:** autophagy, drug development, network pharmacology, oral squamous cell carcinoma, paeonol

## Abstract

**Background:**

Paeonol (Pae), a phenolic bioactive compound extracted from Cortex Moutan, exhibits numerous pharmacological properties, including anti-inflammatory, immunomodulatory, and antitumor activities. However, the precise mechanisms by which Pae influences protective autophagy in oral squamous cell carcinoma (OSCC) remain incompletely characterized.

**Methods:**

This study assessed the effects of Pae treatment on proliferation, migration, and invasive potential of OSCC cells *in vitro*. Network pharmacology was employed to identify potential molecular targets of Pae involved in OSCC. Autophagic flux was analyzed using transmission electron microscopy alongside a dual-fluorescence reporter assay. Additionally, the combined effects of Pae with autophagy inhibitors were investigated.

**Results:**

Pae treatment promoted mitochondrial-dependent apoptosis and effectively inhibited epithelial–mesenchymal transition (EMT) by attenuating phosphorylation within the PI3K/AKT signaling pathway. Pae simultaneously initiated protective autophagy, confirmed by intact autophagic flux observed in CAL-27 and HSC-3 cells. Interference with this autophagic process through the autophagy inhibitor 3-methyladenine (3-MA) intensified apoptotic activity and markedly reduced OSCC cell proliferation.

**Conclusion:**

Pae suppressed OSCC cell proliferation and EMT and was associated with mitochondrial apoptosis and activation of autophagic flux, accompanied by reduced PI3K/AKT phosphorylation. Co-treatment with 3-methyladenine (3-MA) further decreased cell viability and enhanced apoptosis-associated changes, suggesting that pharmacological co-targeting of PI3K signaling and autophagy may potentiate Pae’s antitumor activity. Further studies are warranted to delineate the relative contributions of apoptosis and autophagy to Pae-induced cytotoxicity in OSCC.

## 1 Introduction

Squamous cell carcinoma of the head and neck (SCCHN) ranks sixth globally in terms of cancer prevalence (Machiels et al., 2020). OSCC, recognized as the most aggressive and predominant subtype of SCCHN (Fan et al., 2022), demonstrates high rates of metastasis and recurrence, leading to poor prognostic outcomes and a 5-year survival rate below 50% (Ren et al., 2020; Sung et al., 2021). Current OSCC treatment typically involves surgical resection combined with radiotherapy and chemotherapy, with specific strategies tailored to the tumor’s location and stage. However, existing therapies often lead to severe side effects, such as bone marrow suppression, immunosuppression, and drug resistance. Therefore, the identification of novel agents with potent anti-tumor activity and minimal toxicity is crucial. Natural plant-derived compounds, known for their multi-target mechanisms and favorable safety profiles, represent a promising avenue for such therapeutic advancements (Singh et al., 2016).

Autophagy represents a lysosome-dependent cellular degradation mechanism critical for clearing defective intracellular organelles and pathogens, thereby preserving cellular stability and viability (Deng et al., 2019; Hwang et al., 2019; Harsha et al., 2020). While autophagy can serve as a barrier to cancer progression in early tumorigenesis, it can paradoxically promote tumor cell survival and proliferation in advanced malignancies. Consequently, the inhibition of autophagy has become an attractive therapeutic avenue for cancer intervention (Mizushima and Komatsu, 2011). Acting as a dual-faceted regulatory process in cancer, autophagy serves both as a homeostatic and stress-responsive mechanism (Duan H et al., 2025; Gou et al., 2022). Basal autophagic activity contributes to genomic stability; however, established tumor cells often activate protective autophagy to sustain survival, facilitating therapeutic resistance (Ying et al., 2025). Aberrant activation of the PI3K/AKT/mTOR pathway frequently occurs in OSCC, profoundly affecting tumor cell proliferation, apoptosis, autophagy, and growth, and correlates strongly with aggressive tumor behavior and poor clinical outcomes (Liu et al., 2022; Zhang et al., 2025). EMT, another crucial biological process implicated in OSCC malignancy, contributes significantly to tumor invasiveness and metastatic potential, serving as a key malignancy indicator (Lamouille et al., 2014; Mittal, 2018). Cross-talk between autophagy and EMT has been demonstrated in numerous malignancies, including hepatocellular carcinoma, breast cancer, and melanoma, where autophagy has been shown to facilitate metastasis (Gundamaraju et al., 2022). Autophagy inhibitors such as 3-MA, targeting the PI3K axis to inhibit autophagy, have emerged as vital investigative tools for elucidating the intricate roles of autophagy in cancer biology and treatment (Kaizuka et al., 2016). Previous research has shown that Pae stimulates protective autophagy in ovarian cancer (OC) cells (Gao et al., 2019), resulting in improved antitumor effects when combined with autophagy inhibitors in animal models. This suggests that Pae-induced autophagy may modulate therapeutic response and could be leveraged in combination strategies. However, it remains uncertain if Pae specifically mediates autophagy and apoptosis through the PI3K/AKT pathway in OSCC cells to suppress tumor growth and whether combining autophagy inhibitors enhances these cellular effects.

Natural compounds have drawn increasing research interest as potential therapeutic options for diverse diseases due to their extensive biological activities and unique chemical diversity (Chang et al., 2023). Pae, a phenolic compound obtained from the root bark of the tree peony, exhibits numerous pharmacological activities, including cardioprotective, and anticancer properties (Lv et al., 2022; Zhang et al., 2020). Previous studies confirmed Pae’s effectiveness in inhibiting cell proliferation, promoting apoptosis, and inducing protective autophagy across various cancer types, via multiple molecular targets and pathways (Cheng et al., 2020; Gao et al., 2019; Li, 2010). Moreover, Pae has demonstrated protective properties against OSCC induced by 7,12-dimethylbenz(a)anthracene (Ramachandhiran et al., 2019). Recent findings also indicate that Pae can suppress glycolysis and cell migration in OSCC by inhibiting NAT10-mediated ac4C modification of HK2 (Yang et al., 2025). Nevertheless, the precise molecular mechanisms by which Pae inhibits OSCC cell invasion and migration have yet to be thoroughly clarified. Additionally, the potential regulatory effects of Pae on autophagy and its functional role in OSCC have not been explored.

To address this research gap, the current study utilized an integrated approach combining *in vitro* validation and network pharmacology-based predictions to examine Pae’s role in regulating proliferation, apoptosis, migration, invasion, and autophagy in OSCC cells. Furthermore, this work aimed to explore the participation of the PI3K/AKT signaling axis in Pae-induced autophagy, as well as characterize the features of autophagy triggered by Pae and evaluate the synergistic anticancer efficacy of Pae when combined with the autophagy inhibitor 3-MA. This research not only advances understanding of the mechanisms underlying Pae’s anti-OSCC activity—particularly its modulation of autophagy via the PI3K/AKT pathway—but also provides a robust experimental and theoretical foundation for developing innovative therapeutic strategies for OSCC based on Pae, either alone or in combination with autophagy inhibitors.

## 2 Materials and methods

### 2.1 Chemicals and reagents

We purchased Pae from MedChemExpress (MCE, USA) with a purity level of at least 98%. We dissolved it in dimethyl sulfoxide (DMSO; Solarbio, Beijing) at 200 mmol/L and kept it at −20 °C. In addition, 3-methyladenine (3-MA) and the Cell Counting Kit-8 (CCK-8; Multisciences Biotech, China) were utilized as reagents (MedChemExpress, USA). The protein phosphatase inhibitor and phenylmethylsulfonyl fluoride were purchased from Solarbio in Beijing, China, for the purpose of protein extraction. Affinity provided the PI3K and phosphorylated PI3K antibodies at a 1:1000 dilution, HuaBio provided the AKT and phosphorylated AKT antibodies at a 1:500 dilution, and Abcam provided the Bax, Caspase-3, and Bcl-2 antibodies at 1:2000 dilutions. In addition, MedChemExpress (USA) supplied the cell-permeable PI3K activator 740Y-P (HY-P0175).

### 2.2 Cell culture

Short tandem repeat (STR) profiling was used for verification of OSCC cell lines CAL-27 and HSC-3, as well as normal oral keratinocytes (NOK). The cells were grown in a humidified environment at 37 °C with 5% CO_2_ in Dulbecco’s Modified Eagle Medium (DMEM) that contained 10% fetal bovine serum (FBS) and 1% penicillin-streptomycin. In every experiment, cells were employed while they were in the logarithmic growth phase.

### 2.3 Cell Counting kit-8 (CCK-8) assay

A density of 5 × 10^3^ cells per well of CAL-27, HSC-3, and NOK cells was used in the seeding of 96-well plates, which were then incubated at 37 °C for 24 h. Cells were then treated for an additional 24 h with several doses of Pae (0.5, 1, 1.5, 2, 2.5, and 3 mM) or a vehicle control (DMSO). The maximum concentration of Pae was mimicked in the DMSO concentration given to control cells. Three times, the experiments were repeated. After incubating with a 10% CCK-8 solution for 2 h, the absorbance at 450 nm was measured using a microplate reader to test cell viability. Thus, half-maximal inhibitory concentration (IC_50_) values were computed, and Pae concentrations of 0, 0.4, 0.8, and 1.6 mmol/L were chosen for the following experiments according to these findings.

### 2.4 Scratching assay

Standard conditions were used to incubate CAL-27 and HSC-3 cells in 6-well plates with 2 mL of DMEM supplemented with 10% FBS until around 90% confluence was achieved. A sterile pipette tip was used to create uniform wounds. Cells were washed with phosphate-buffered saline (PBS) and then cultured in new media with Pae (0, 0.4, 0.8, 1.6 mM) after first imaging under an inverted microscope. The wound healing rates were determined by taking images again at 24 h and dividing the difference between the scratch areas at 0 and 24 h by the scratch areas at 0 h, and then multiplying the result by 100%.

### 2.5 Transwell assay

The upper chambers of the transwell inserts were coated with 200 μL of Matrigel that had been diluted in serum-free DMEM at a 1:8 ratio. The coated inserts were planted with CAL-27 and HSC-3 cells that were suspended in serum-free DMEM with Pae (0, 0.4, 0.8, 1.6 mM). The chemoattractant in the lower chambers was 500 μL of DMEM containing 10% FBS. The invasive cells were preserved with 4% paraformaldehyde after 24 h of incubation, stained with 0.1% crystal violet, and gently washed with PBS. Using cotton swabs, non-invasive cells were extracted. To measure the number of invasive cells, images were taken from five fields at a magnification of ×200, which were chosen at random.

### 2.6 Cell colony formation assay

CAL-27 and HSC-3 cells were placed in 6-well plates at 1 × 10^3^ cells/well and left to adhere for a full day. The culture was maintained for a total of 14 days after exposure to the stated treatments, with three duplicates per condition. Media was replaced every 3 days thereafter. To assess their clonogenic capacity, cells were washed with PBS, fixed with 4% formaldehyde for 20 min, stained with 0.1% crystal violet for 30 min, and finally counted.

### 2.7 Western blot (WB)

Following 24 h of culture, the cells were subjected to total protein extraction using ice-cold RIPA buffer that had been treated with PMSF and protein phosphatase inhibitors (Solarbio, Beijing). A BCA assay kit (Solarbio) was used to quantify protein quantities. Proteins in equal concentrations were denaturated at 100 °C, separated using 10% SDS-PAGE, and then transferred to PVDF membranes. After being coated with Rapid Closure Solution for 20 min, the membranes were incubated overnight with primary antibodies (with β-actin or GAPDH as loading controls; for EMT-related blots, total-protein staining was used as an additional loading verification). The next day, they were incubated under dark conditions with secondary antibodies. Using an ECL detection system, proteins were seen, and band intensities were evaluated using ImageJ software.

### 2.8 Pae target prediction

Structural data of Pae (SDF format) were acquired from PubChem and subsequently uploaded to the Pharmmapper platform to predict potential molecular targets. Resultant protein targets were standardized into gene symbols utilizing the UniProt database.

### 2.9 Disease target prediction

OSCC-related molecular targets were identified by querying the DisGeNET (https://www.disgenet.org/), GeneCards (https://www.genecards.org/), and OMIM (https://omim.org/) databases using the keyword “OSCC.” After consolidating the results, duplicate entries were removed to compile a comprehensive list of OSCC-associated targets.

### 2.10 Acquisition of pae targets with OSCC intersection targets

The intersection between Pae and OSCC-related targets was established, and overlapping targets were visualized through a Venn diagram constructed using the online Microbiotics tool (http://www.bioinformatics.com.cn/?p=1).

### 2.11 Protein-protein interaction (PPI) network construction

Overlapping targets were submitted to the STRING database (https://cn.string-db.org/) with the organism set to “*Homo sapiens*” and a minimum required interaction score of 0.4 (medium confidence). The resulting protein-protein interaction (PPI) network was exported and imported into Cytoscape (v3.7.2) for visualization and topological analysis. Hub targets were ranked using the CytoHubba plugin based on the maximal clique centrality (MCC) algorithm; nodes were sorted by MCC score, and the top 10 targets were defined as hub genes.

### 2.12 GO and KEGG pathway enrichment analyses

The DAVID database (https://david.ncifcrf.gov/) was used to conduct enrichment analysis on overlapping targets. The significance criterion was set at p < 0.05, and the target species chosen was “*H. sapiens*”. Categories that were enhanced comprised GO Biological Processes (BP), Molecular Functions (MF), Cellular Components (CC), and KEGG pathways. With the help of the Weixin web (http://www.bioinformatics.com.cn/?p=1) application, bubble charts were created to show the twenty most enriched KEGG pathways, and bar charts were used to display the ten most important GO keywords (BP, MF, CC).

### 2.13 Apoptosis flow cytometry

Before being treated with different doses of Pae (0, 0.4, 0.8, and 1.6 mM) for 24 h, CAL-27 and HSC-3 cells were plated in 6 cm culture dishes until they reached about 50%–70% confluence. Using an Annexin V-FITC/PI Apoptosis Detection Kit (KGA1102, KeyGenBio, China), cells were detached using EDTA-free trypsin and subsequently stained upon collection. Flow cytometry (FC) was used to quantify apoptotic cells, and the data were processed with the help of FlowJo software.

### 2.14 Transmission electron microscope

Centrifugation was used to extract CAL-27 and HSC-3 cells after they had been treated with Pae (1.6 mM) for 24 h. The cells were then fixed overnight with 2.5% glutaraldehyde. The cells were first immersed in 1% agarose, then fixed for 2 h at room temperature in darkness using 1% osmium tetroxide. After that, they were dehydrated using an ethanol gradient and embedded in acetone. Representative micrographs were taken after staining and examining ultrathin slices (60–80 nm) under a transmission electron microscope.

### 2.15 Autophagic flux analysis

Under typical incubation settings (37 °C, 5% CO_2_, and 90% humidity), CAL-27 cells were transfected with mTagRFP-senseGFP-LC3-lentivirus (KL103481-jlV, KeyGenBio, China). After that, they were treated with Pae (1.6 mM) for 24 h. The autophagic flux was tracked and recorded with the help of a laser confocal microscope (FV3000-RS, Olympus).

### 2.16 Statistical analysis

GraphPad Prism software (version 10) was utilized for statistical evaluation and graphical representation. Data from experiments repeated independently three times were presented as mean ± standard deviation (SD). Appropriate statistical tests, including Student’s t-test, one-way ANOVA, two-way ANOVA, or three-way ANOVA, were employed to determine group differences. Statistical significance was defined at p < 0.05, with significance levels marked as follows: ns (p > 0.05), *p < 0.05, **p < 0.01, ***p < 0.001, and ****p < 0.000.

## 3 Results

### 3.1 Pae inhibited the migration and invasion of oral squamous cell carcinoma (OSCC) cells in a concentration-dependent manner

**FIGURE 1:**
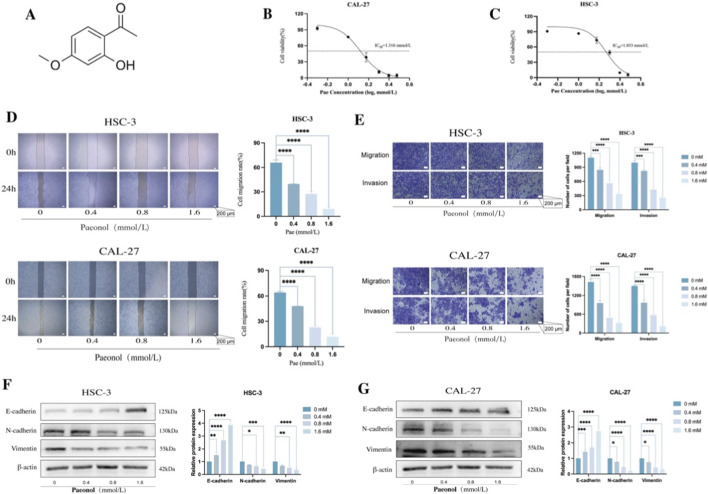
Pae suppresses OSCC migration and invasion and modulates EMT-related protein levels. **(A)** Pae chemical structure. **(B,C)** Viability of CAL-27 and HSC-3 cells post-Pae treatment at indicated concentrations (24 h), evaluated via CCK-8 assay. **(D)** Scratch wound assay images illustrating CAL-27 and HSC-3 cell migration suppression after Pae exposure. Scale bar: 200 μm. **(E)** Transwell assay images demonstrating reduced invasive and migratory activities in Pae-treated OSCC cells. Scale bar: 200 μm. **(F,G)** WB analysis and quantification of E-cadherin, N-cadherin, and Vimentin, showing significant alterations upon Pae treatment.

The chemical structure of Pae is illustrated in Figure 1A. To ascertain optimal dosages, CAL-27 and HSC-3 cells were treated with increasing concentrations of Pae for 24 h. IC_50_ values, assessed using the CCK-8 assay, were identified as 1.853 mmol/L for HSC-3 and 1.316 mmol/L for CAL-27 cells (Figures 1B,C). Subsequent experiments utilized Pae concentrations representing approximately ¼ IC_50_, ½ IC_50_, and full IC_50_ values (0.4, 0.8, and 1.6 mM). A dose-dependent inhibitory effect of Pae on cell migration was observed via scratch wound assays in both OSCC cell lines (Figures 1D,E). Complementary Transwell assays further validated these results, demonstrating significant suppression of cell migration and invasion upon Pae treatment (Figures 1F,G). Since EMT significantly contributes to cancer cell invasiveness and migration (Patra et al., 2020), EMT-related markers (Du and Shim, 2016), and Vimentin were examined using WB. Pae treatment notably increased E-cadherin while reducing N-cadherin and Vimentin, suggesting that Pae effectively suppresses OSCC cell invasiveness and migration.

### 3.2 Pae inhibited the proliferation of OSCC cells in a concentration-dependent manner

**FIGURE 2:**
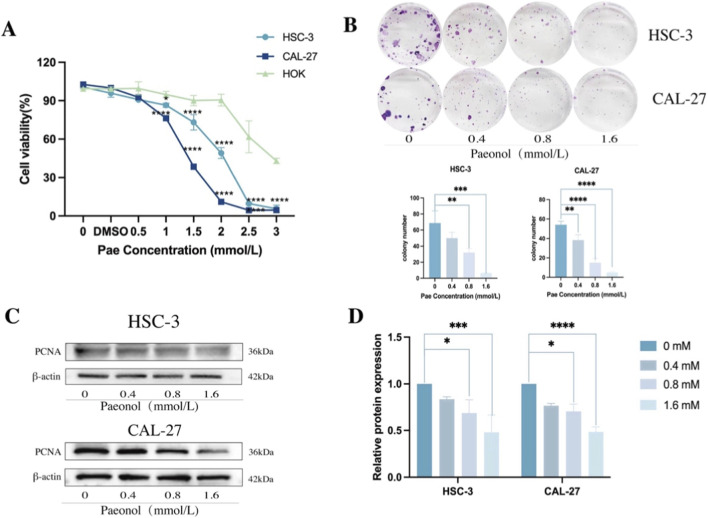
Pae reduces OSCC proliferation and downregulates proliferation-associated protein expression. **(A)** CAL-27, HSC-3, and NOK cell viability after 24 h Pae treatment assessed by CCK-8 assay. **(B)** Colony formation capabilities of CAL-27 and HSC-3 cells exposed to Pae over 14 days **(C,D)** WB analysis and quantitative data showing PCNA expression decline following Pae treatment.

To further investigate the impact of Pae on OSCC cell viability, CAL-27, HSC-3, and NOK were incubated with Pae concentrations ranging from 0 to 3 mM for 24 h. The CCK-8 assay indicated that OSCC cell viability diminished with increasing Pae concentration, while NOK viability remained largely unaffected at concentrations below 2 mmol/L, indicating limited cytotoxic effects on normal epithelial cells (Figure 2A). Colony formation assays further revealed that higher Pae concentrations notably suppressed clonogenic potential in both OSCC cell lines (Figure 2B). WB analysis of proliferating cell nuclear antigen (PCNA) protein showed concentration-dependent decreases after Pae treatment, confirming Pae’s anti-proliferative effects (Figure 2C). These collective results demonstrate the inhibitory capacity of Pae on OSCC proliferation, with minimal cytotoxicity toward normal oral epithelial cells.

### 3.3 Common targets of pae and OSCC

**FIGURE 3:**
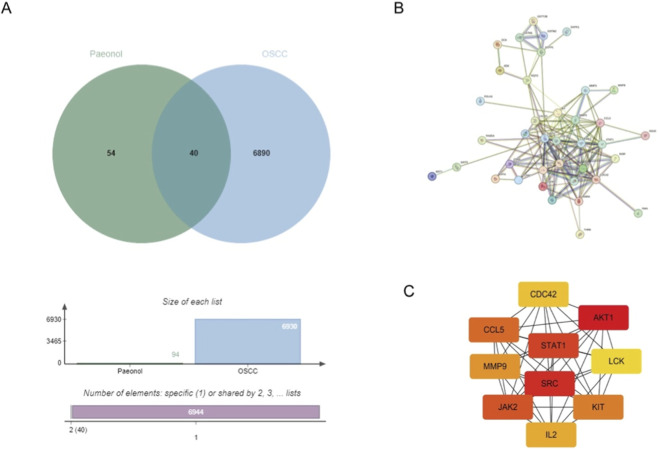
Common targets of Pae and OSCC, PPI network construction, and core target screening. **(A)** Venn diagram of common targets of Pae and OSCC. **(B)** PPI network of potential core targets associated with Pae and OSCC. **(C)** Top 10 core targets in the PPI network.

Based on the PharmMapper database, 95 potential target proteins of Pae were identified. From the GeneCards database, 6107 OSCC-related targets were retrieved, and an additional 1019 targets were sourced from the OMIM database. Following merging and eliminating duplicates, 639 targets related to OSCC were identified. The potential targets of Pae were compared with the OSCC disease targets, revealing 40 common targets. A Venn diagram illustrating this overlap was created utilizing an online application (Figure 3A).

### 3.4 PPI network construction and core target screening

Forty intersecting targets between Pae and OSCC were analyzed further by generating a PPI network via the STRING database (Figure 3B). The generated network comprised 40 nodes (with two proteins showing no interactions) connected by 146 edges, with an average interaction degree of 7.3. Hub targets were ranked using the MCC algorithm in CytoHubba, and the top 10 targets were selected as hub genes (Figure 3C), including CDC42, AKT1, CCL5, STAT1, MMP9, LCK, SRC, JAK2, KIT, and IL2. These results suggest that these proteins may serve as core targets through which Pae exerts its anti-OSCC effects.

### 3.5 GO and KEGG analyses

**FIGURE 4:**
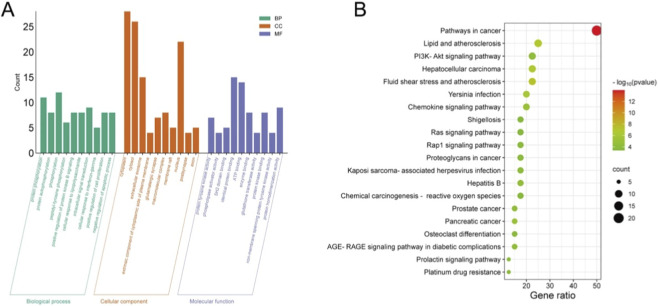
GO and KEGG enrichment analyses. **(A)** GO analysis. **(B)** KEGG analysis bubble plot.

The top ten enriched GO terms within BP, CC, and MF categories are presented in Figure 4A. The predominant BP terms included protein phosphorylation, regulation of protein kinase B signaling, stimulation of cell proliferation, and inhibition of apoptosis. Primary CC terms involved cytosolic regions and macromolecular complexes, while notable MF terms encompassed ATP binding, enzyme binding, protein kinase binding, and phospholipase activator activity. Additionally, the twenty most significant KEGG pathways are depicted in a bubble plot (Figure 4B). Of these, pathways such as PI3K/AKT and chemokine signaling were markedly enriched. Given its significant relevance, the PI3K/AKT pathway was chosen for further exploration.

### 3.6 Pae induces apoptosis of OSCC cells via the PI3K/AKT pathway

**FIGURE 5:**
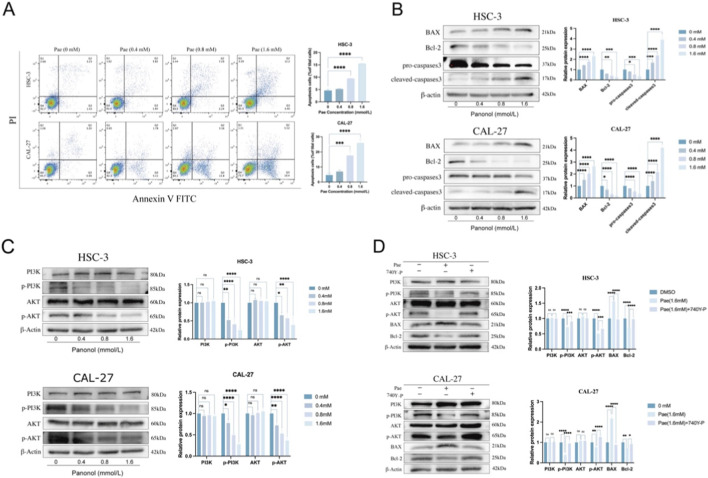
Pae induces apoptosis in OSCC cells via suppression of PI3K/AKT signaling. **(A)** FC analysis of apoptosis in CAL-27 and HSC-3 cells treated with Pae. **(B)** WB quantification of apoptosis-related proteins (Bax, Bcl-2, caspase-3 forms). **(C)** WB evaluation of PI3K/AKT phosphorylation post-Pae treatment. **(D)** Protein expression analysis following combined Pae and PI3K activator treatment.

To clarify whether apoptosis mediated the inhibitory effects of Pae on OSCC proliferation, apoptosis was analyzed via Annexin V/PI staining and FC. Pae exposure increased the proportions of early and late apoptotic cells in a dose-dependent manner (Figure 5A). Additionally, WB results demonstrated marked downregulation of Bcl-2 and upregulation of Bax and cleaved-caspase-3, accompanied by reduced pro-caspase-3 levels in Pae-treated cells compared to untreated controls (Figure 5B). Network pharmacology analyses identified the PI3K/AKT pathway as a potential apoptotic mechanism targeted by Pae. Consistently, Pae reduced the phosphorylation of PI3K and AKT (p-PI3K, p-AKT) without altering their total protein expression levels (Figure 5C). Further validation through PI3K activation demonstrated reversal of Pae-induced changes in PI3K/AKT phosphorylation and apoptosis-associated proteins upon activator co-treatment (Figure 5D).

### 3.7 Pae induces autophagy in OSCC cells

**FIGURE 6:**
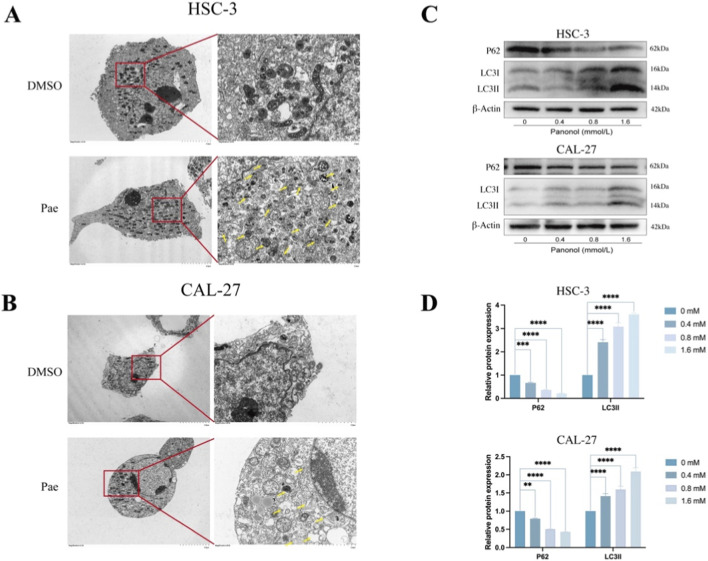
Pae enhances autophagic activity in OSCC cells. **(A,B)** TEM images showing increased autophagic structures post-Pae treatment (24 h, 1.6 mM). **(C,D)** WB quantification of LC3-II and p62 protein levels following Pae exposure.

Autophagy induction by Pae was analyzed through transmission electron microscopy (TEM) in OSCC cells after 1.6 mM Pae exposure for 24 h. TEM images revealed increased autolysosomal structures in treated cells compared to controls (Figures 6A,B). Autophagic activity was further examined by assessing autolysosomal structures, which were notably increased in Pae-treated cells compared to controls (Liu et al., 2023). WB analysis of autophagy markers p62 and LC3 showed decreased p62 and elevated LC3-I / LC3-II levels dose-dependently after Pae treatment, reinforcing the induction of autophagy in OSCC cells (Figures 6C,D).

### 3.8 Pae activates autophagic flux in OSCC cells

**FIGURE 7:**
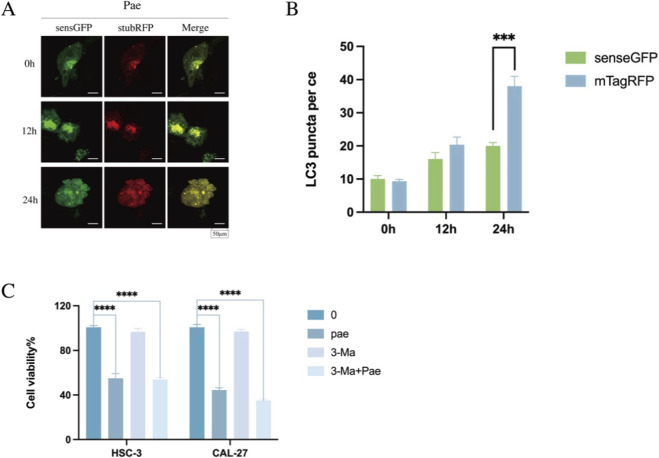
Pae-induced autophagic flux is enhanced by autophagy inhibition in OSCC cells. **(A,B)** CAL-27 cells transfected with mTagRFP-senseGFP-LC3 were exposed to Pae (1.6 mM) for 0, 12, and 24 h and observed using confocal microscopy. Scale bar = 50 μm. **(C)** OSCC cell viability assessed by CCK-8 assay after treatments.

During autophagosome formation, LC3-I is converted to its lipidated form LC3-II, which serves as an established marker for autophagy (Zhou et al., 2012). To investigate Pae-induced autophagic flux comprehensively, CAL-27 cells were transduced with mTagRFP-senseGFP-LC3 lentivirus. The SensGFP fluorescent signal diminishes under the acidic environment of autolysosomes, whereas StubRFP fluorescence remains stable irrespective of pH (Kaizuka et al., 2016; Singh et al., 2014). This dual-fluorescence system was used to assess autophagic flux (Figures 7A,B). Compared to untreated controls, CAL-27 cells treated with 1.6 mM Pae for 24 h showed numerous yellow puncta (StubRFP^+^/SensGFP^+^), indicating the presence of autophagosomes. Notably, red puncta (StubRFP^+^) increased significantly, while green fluorescence (SensGFP^+^) decreased after Pae treatment, reflecting the formation of autolysosomes. Confocal microscopy revealed elevated accumulation of autophagosomes and autolysosomes following Pae treatment, confirming enhanced autophagic flux. These observations align closely with the WB results, providing additional support for Pae’s autophagy-promoting effects in OSCC cells.

### 3.9 Pharmacological co-treatment with 3-MA potentiates Pae-mediated growth inhibition and apoptosis-associated changes

**FIGURE 8:**
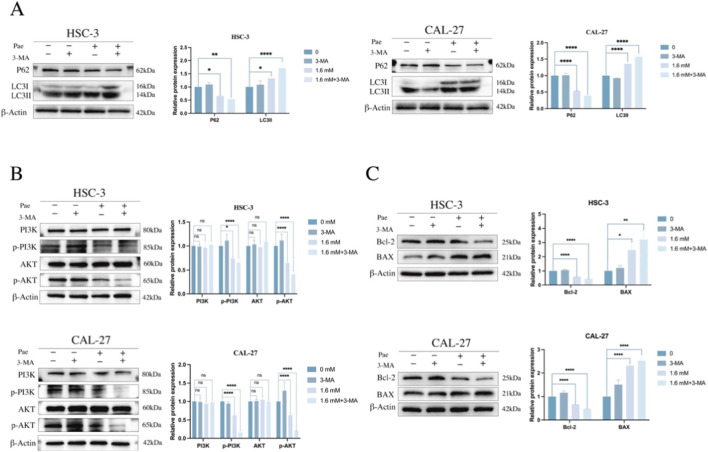
The autophagy inhibitor 3-MA potentiates Pae-induced antiproliferative and apoptotic responses in OSCC cells. **(A)** WB and quantification of LC3 and p62 expression in OSCC cells pretreated with 3-MA (2.5 mM, 1 h) followed by Pae (1.6 mM, 24 h). **(B)** WB analysis and densitometric quantification of phosphorylated and total PI3K and AKT protein expression post-treatment. **(C)** Expression levels of apoptosis-related proteins Bcl-2 and Bax after combined treatment.

To examine whether pharmacological modulation of autophagy/PI3K signaling influences the cellular response to Pae, OSCC cells were treated with 3-MA, an inhibitor of class III PI3K commonly used to suppress early-stage autophagy. The CCK-8 assay showed that co-treatment with Pae (1.6 mM) and 3-MA (2.5 mM) reduced cell viability more than Pae alone (Figure 7C). In WB analyses, 3-MA alone increased p62 and reduced LC3-I/LC3-II, whereas co-treatment altered LC3-I/LC3-II and p62 levels relative to Pae alone (Figure 8A), consistent with modulation of the autophagy pathway. Co-treatment also decreased p-PI3K and p-AKT (Figure 8B) and shifted apoptosis-related proteins toward a pro-apoptotic pattern (decreased Bcl-2 and increased Bax; Figure 8C). Collectively, these data indicate that 3-MA co-treatment potentiates Pae-associated growth inhibition and apoptosis-related changes, although the relative contributions of autophagy inhibition versus PI3K pathway effects require further clarification.

## 4 Discussion

OSCC progression is significantly driven by a dual mechanism involving tumor cell proliferation and invasiveness, posing challenges due to high invasiveness, frequent recurrence, and limited therapeutic options. Uncontrolled proliferation is closely linked to the aberrant activation of the PI3K/AKT/mTOR pathway (Harsha et al., 2020), while invasive capabilities are significantly enhanced via activation of EMT (Nieto et al., 2016; Allgayer et al., 2025; Qu et al., 2023). In recent years, natural phytochemicals have gained recognition as promising adjuncts for cancer therapy due to their antitumor properties, low toxicity, and favorable safety profile (Chang et al., 2023; Wu et al., 2024). Pae, a pharmacologically active compound isolated from Cortex Moutan, has demonstrated significant anticancer properties, primarily via apoptosis activation and suppression of angiogenesis in several malignancies, such as esophageal (Duan Y et al., 2025), bladder (Ying et al., 2025), ovarian (Gao et al., 2019), and lung cancers (Yan et al., 2025). Moreover, Pae derivatives exhibit promising antitumor activity, offering crucial structural templates for novel anticancer drug discovery (Tsai et al., 2016). In our present research, Pae substantially inhibited OSCC cell proliferation, invasive capacity, and migration through mechanisms involving apoptosis enhancement and suppression of the PI3K/AKT pathway. Notably, Pae also activated protective autophagy, and combining Pae with the autophagy inhibitor 3-MA disrupted this protective mechanism, enhancing Pae’s antitumor efficacy.

This study further shows that Pae-induced mitochondrial apoptosis is associated with reduced phosphorylation within the PI3K/AKT signaling axis, reflected by elevated Bax/Bcl-2 ratios and increased caspase-3 activation. These findings align well with those previously reported by Gao and colleagues in the context of OC (Gao et al., 2019). The intrinsic apoptosis pathway is modulated by Bcl-2 family proteins (Tait and Green, 2010; Cory and Adams, 2002), encompassing both pro-apoptotic proteins such as Bax and anti-apoptotic proteins. Additionally, apoptosis and autophagy represent closely interlinked processes that collectively regulate cellular equilibrium and survival decisions, with growing evidence highlighting their reciprocal regulation (Sorice, 2022; Yonekawa and Thorburn, 2013). Numerous reports have underscored the importance of the PI3K/AKT cascade in regulating apoptosis, autophagy, and tumor cell survival (Fulda, 2014; Tong et al., 2022; Mei et al., 2025). Notably, inhibition of the PI3K/AKT pathway can alleviate mTOR-mediated suppression of autophagy and promote autophagic flux. In our study, the mTagRFP-senseGFP-LC3 dual-fluorescence system revealed normal conversion of autophagosomes to autolysosomes, indicating intact autophagic degradation. Pae-induced autophagy in OSCC appears to function as a cytoprotective response: co-treatment with 3-MA further reduced cell viability and enhanced apoptosis-associated changes. Nevertheless, pharmacological agents such as 3-MA may exert pathway effects beyond autophagy, and genetic or rescue approaches will be required to define causality.

Combining autophagy inhibitors and paclitaxel has been explored as a therapeutic strategy in advanced pancreatic ductal carcinoma (Karasic et al., 2019). In our study, co-treatment with Pae and 3-MA enhanced apoptosis-associated changes and further reduced OSCC cell viability. Although Pae induced intact autophagic flux, the changes in LC3-I / LC3-II and p62 observed under 3-MA co-treatment are interpreted as pharmacological modulation of the autophagy pathway rather than definitive evidence of “autophagic collapse”. Given that 3-MA targets PI3K and may influence additional signaling cascades, the contribution of autophagy inhibition versus other pathway effects requires further clarification (Basit et al., 2017).

The PI3K/AKT pathway functions as a crucial regulatory node that can influence apoptosis, autophagy, and EMT. In our experiments, Pae decreased PI3K/AKT phosphorylation and activated mitochondrial apoptosis markers. Pae also induced autophagic flux, and 3-MA co-treatment further reduced cell viability and enhanced apoptosis-associated changes. The mechanistic links among PI3K/AKT inhibition, autophagy modulation, and EMT suppression warrant future investigation using genetic and rescue approaches to dissect pathway dependencies and exclude contributions from other regulated cell death programs (Shao et al., 2019).

**FIGURE 9:**
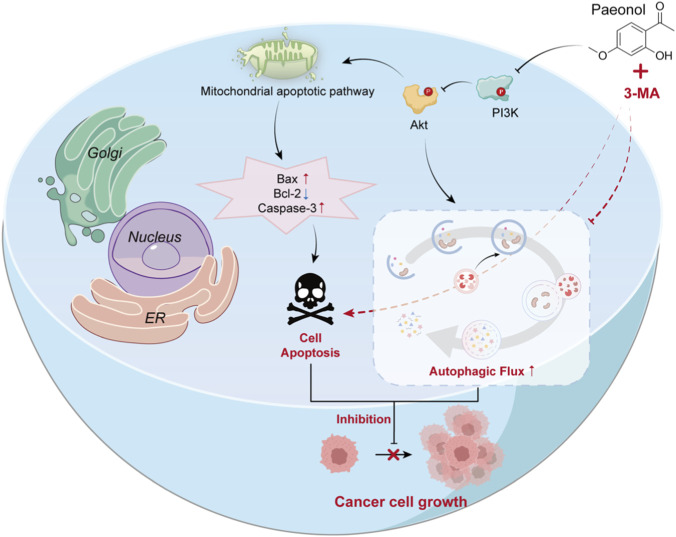
Mechanism of development in oral squamous cell carcinoma cells by Pae.

To our knowledge, this study is the first to report that Pae simultaneously regulates apoptosis, protective autophagy, and EMT via the PI3K/AKT pathway in OSCC cells (Liang et al., 2021), thus challenging the traditional characterization of this pathway as exclusively pro-survival. Pae not only induces apoptosis and inhibits EMT-related processes but also maintains autophagic flux, indicating a complex mechanism underlying its anticancer activity. The complete autophagic flux in HSC-3 and CAL-27 cells was further verified using the mRFP-GFP-LC3 dual-fluorescent system. Importantly, blocking protective autophagy by adding 3-MA markedly augmented apoptosis and reduced cellular viability, highlighting the synergistic therapeutic potential of this combined treatment strategy. This discovery offers a novel perspective for understanding the multi-target mechanism underlying Pae’s anti-OSCC activity and proposes a new strategy for overcoming chemotherapy resistance in clinical settings: a low-toxicity synergistic approach based on Pae combined with autophagy inhibitors. This strategy enables more effective OSCC treatment by concurrently targeting three key BP—apoptosis, autophagy, and EMT. An abstract representation of the article is illustrated in Figure 9.

## 5 Conclusion

Collectively, our results show that Pae inhibits OSCC cell proliferation, migration/invasion, and EMT, accompanied by reduced PI3K/AKT phosphorylation, activation of mitochondrial apoptosis markers, and induction of intact autophagic flux. Co-treatment with 3-MA further potentiated growth inhibition and apoptosis-associated changes. A key limitation is that we did not perform genetic modulation of autophagy (e.g., ATG5/ATG7 silencing) or apoptosis rescue (e.g., pan-caspase inhibition) to quantify the relative contributions of apoptosis and autophagy to Pae-induced cytotoxicity or to exclude other regulated cell death pathways. These experiments, together with *in vivo* validation and safety assessment of the combination strategy, will be addressed in future studies.

## Data Availability

The datasets presented in this study can be found in online repositories. The names of the repository/repositories and accession number(s) can be found below: https://pubchem.ncbi.nlm.nih.gov/, 11092.

## References

[B1] Allgayer H. Mahapatra S. Mishra B. Swain B. Saha S. Khanra S. (2025). Epithelial-to-mesenchymal transition (EMT) and cancer metastasis: the status quo of methods and experimental models 2025. Mol. Cancer 24 (1), 167. 10.1186/s12943-025-02338-2 40483504 PMC12144846

[B2] Basit F. Van Oppen L. M. Schöckel L. Bossenbroek H. M. van Emst-de Vries S. E. Hermeling J. C. (2017). Mitochondrial complex I inhibition triggers a mitophagy-dependent ROS increase leading to necroptosis and ferroptosis in melanoma cells. Cell Death Dis. 8 (3), e2716. 10.1038/cddis.2017.133 28358377 PMC5386536

[B3] Chang X. Feng X. Du M. Li S. Wang J. Wang Y. (2023). Pharmacological effects and mechanisms of paeonol on antitumor and prevention of side effects of cancer therapy. Front. Pharmacol. 14, 1194861. 10.3389/fphar.2023.1194861 37408762 PMC10318156

[B4] Cheng C. S. Chen J. X. Tang J. Geng Y. W. Zheng L. Lv L. L. (2020). Paeonol inhibits pancreatic cancer cell migration and invasion through the inhibition of TGF-β1/Smad signaling and epithelial-mesenchymal-transition. CMAR 12, 641–651. 10.2147/CMAR.S224416 32099461 PMC6996112

[B5] Cory S. Adams J. M. (2002). The Bcl2 family: regulators of the cellular life-or-death switch. Nat. Rev. Cancer 2 (9), 647–656. 10.1038/nrc883 12209154

[B6] Deng S. Shanmugam M. K. Kumar A. P. Yap C. T. Sethi G. Bishayee A. (2019). Targeting autophagy using natural compounds for cancer prevention and therapy. Cancer 125 (8), 1228–1246. 10.1002/cncr.31978 30748003

[B7] Du B. Shim J. (2016). Targeting epithelial–mesenchymal transition (EMT) to overcome drug resistance in cancer. Molecules 21 (7), 965. 10.3390/molecules21070965 27455225 PMC6273543

[B8] Duan H H. Li Y. Zheng X. Hou J. Tao H. Liu X. (2025). Paeonol enhances a recombinant EGFR-targeted fusion protein-drug conjugate induced antitumor efficacy in esophageal cancer. Biochem. Pharmacol. 236, 116856. 10.1016/j.bcp.2025.116856 40054783

[B9] Duan Y Y. Yao R. qi Ling H. Zheng L. Y. Fan Q. (2025). Organellophagy regulates cell death:a potential therapeutic target for inflammatory diseases. J. Adv. Res. 70, 371–391. 10.1016/j.jare.2024.05.012 38740259 PMC11976430

[B10] Fan T. Wang X. Zhang S. Deng P. Jiang Y. Liang Y. (2022). NUPR1 promotes the proliferation and metastasis of oral squamous cell carcinoma cells by activating TFE3-dependent autophagy. Sig Transduct. Target Ther. 7 (1), 130. 10.1038/s41392-022-00939-7 35462576 PMC9035452

[B11] Fulda S. (2014). Synthetic lethality by co-targeting mitochondrial apoptosis and PI3K/Akt/mTOR signaling. Mitochondrion 19, 85–87. 10.1016/j.mito.2014.04.011 24780492

[B12] Gao L. Wang Z. Lu D. Huang J. Liu J. Hong L. (2019). Paeonol induces cytoprotective autophagy *via* blocking the Akt/mTOR pathway in ovarian cancer cells. Cell Death Dis. 10 (8), 609. 10.1038/s41419-019-1849-x 31406198 PMC6690917

[B13] Gou Q. Zheng L. L. Huang H. (2022). Unravelling the roles of Autophagy in OSCC: a renewed perspective from mechanisms to potential applications. Front. Pharmacol. 13, 994643. 10.3389/fphar.2022.994643 36263139 PMC9574005

[B14] Gundamaraju R. Lu W. Paul M. K. Jha N. K. Gupta P. K. Ojha S. (2022). Autophagy and EMT in cancer and metastasis: who controls whom? Biochimica Biophysica Acta (BBA) - Mol. Basis Dis. 1868 (9), 166431. 10.1016/j.bbadis.2022.166431 35533903

[B15] Harsha C. Banik K. Ang H. L. Girisa S. Vikkurthi R. Parama D. (2020). Targeting AKT/mTOR in oral cancer: mechanisms and advances in clinical trials. IJMS 21 (9), 3285. 10.3390/ijms21093285 32384682 PMC7246494

[B16] Hwang S. T. Kim C. Lee J. H. Chinnathambi A. Alharbi S. A. Shair O. H. M. (2019). Cycloastragenol can negate constitutive STAT3 activation and promote paclitaxel-induced apoptosis in human gastric cancer cells. Phytomedicine 59, 152907. 10.1016/j.phymed.2019.152907 30981183

[B17] Kaizuka T. Morishita H. Hama Y. Tsukamoto S. Matsui T. Toyota Y. (2016). An autophagic flux probe that releases an internal control. Mol. Cell 64 (4), 835–849. 10.1016/j.molcel.2016.09.037 27818143

[B18] Karasic T. B. O’Hara M. H. Loaiza-Bonilla A. Reiss K. A. Teitelbaum U. R. Borazanci E. (2019). Effect of gemcitabine and nab-Paclitaxel with or without hydroxychloroquine on patients with advanced pancreatic cancer: a phase 2 randomized clinical trial. JAMA Oncol. 5 (7), 993–998. 10.1001/jamaoncol.2019.0684 31120501 PMC6547080

[B19] Lamouille S. Xu J. Derynck R. (2014). Molecular mechanisms of epithelial–mesenchymal transition. Nat. Rev. Mol. Cell Biol. 15 (3), 178–196. 10.1038/nrm3758 24556840 PMC4240281

[B20] Li N. Fan L. L. Sun G. P. Wan X. A. Wang Z. G. Wu Q. (2010). Paeonol inhibits tumor growth in gastric cancer *in vitro* and *in vivo* . WJG 16 (35), 4483–4490. 10.3748/wjg.v16.i35.4483 20845518 PMC2941074

[B21] Liang S. Guo H. Ma K. Li X. Wu D. Wang Y. (2021). A PLCB1–PI3K–AKT signaling axis activates EMT to promote cholangiocarcinoma progression. Cancer Res. 81 (23), 5889–5903. 10.1158/0008-5472.CAN-21-1538 34580062 PMC9397629

[B22] Liu W. Jin W. Zhu S. Chen Y. Liu B. (2022). Targeting regulated cell death (RCD) with small-molecule compounds in cancer therapy: a revisited review of apoptosis, autophagy-dependent cell death and necroptosis. Drug Discov. Today 27 (2), 612–625. 10.1016/j.drudis.2021.10.011 34718209

[B23] Liu S. Yao S. Yang H. Liu S. Wang Y. (2023). Autophagy: regulator of cell death. Cell Death Dis. 14 (10), 648. 10.1038/s41419-023-06154-8 37794028 PMC10551038

[B24] Lv J. Zhu S. Chen H. Xu Y. Su Q. Yu G. (2022). Paeonol inhibits human lung cancer cell viability and metastasis *in vitro via* miR ‐126‐5p/ZEB2 axis. Drug Dev. Res. 83 (2), 432–446. 10.1002/ddr.21873 34636432

[B25] Machiels J. P. René Leemans C. Golusinski W. Grau C. Licitra L. Gregoire V. (2020). Squamous cell carcinoma of the oral cavity, larynx, oropharynx and hypopharynx: EHNS–ESMO–ESTRO Clinical Practice Guidelines for diagnosis, treatment and follow-up. Ann. Oncol. 31 (11), 1462–1475. 10.1016/j.annonc.2020.07.011 33239190

[B26] Mei W. Wei M. Tang C. Li W. Ye B. Xin S. (2025). BCAT2 binding to PCBP1 regulates the PI3K/AKT signaling pathway to inhibit autophagy-related apoptosis and ferroptosis in prostate cancer. Cell Death Dis. 16 (1), 337. 10.1038/s41419-025-07559-3 40274762 PMC12022009

[B27] Mittal V. (2018). Epithelial mesenchymal transition in tumor metastasis. Annu. Rev. Pathol. Mech. Dis. 13 (1), 395–412. 10.1146/annurev-pathol-020117-043854 29414248

[B28] Mizushima N. Komatsu M. (2011). Autophagy: renovation of cells and tissues. Cell 147 (4), 728–741. 10.1016/j.cell.2011.10.026 22078875

[B29] Nieto M. A. Huang R. Y. J. Jackson R. A. Thiery J. P. (2016). EMT: 2016. Cell 166 (1), 21–45. 10.1016/j.cell.2016.06.028 27368099

[B30] Patra S. Panda P. K. Naik P. P. Panigrahi D. P. Praharaj P. P. Bhol C. S. (2020). Terminalia bellirica extract induces anticancer activity through modulation of apoptosis and autophagy in oral squamous cell carcinoma. Food Chem. Toxicol. 136, 111073. 10.1016/j.fct.2019.111073 31877368

[B31] Qu Y. He Y. Wang Y. Han Z. Qin L. (2023). Targeted down-regulation of SRSF1 exerts anti-cancer activity in OSCC through impairing lysosomal function and autophagy. iScience 26 (12), 108330. 10.1016/j.isci.2023.108330 38025785 PMC10663830

[B32] Ramachandhiran D. Vinothkumar V. Babukumar S. (2019). Paeonol exhibits anti-tumor effects by apoptotic and anti-inflammatory activities in 7,12-dimethylbenz(a)anthracene induced oral carcinogenesis. Biotech. and Histochem. 94 (1), 10–25. 10.1080/10520295.2018.1493221 30101628

[B33] Ren Z. Hu C. He H. Li Y. Lyu J. (2020). Global and regional burdens of oral cancer from 1990 to 2017: results from the global burden of disease study. Cancer Commun. 40 (2-3), 81–92. 10.1002/cac2.12009 32067418 PMC7163731

[B34] Shao Q. Wang Q. Wang J. (2019). LncRNA SCAMP1 regulates ZEB1/JUN and autophagy to promote pediatric renal cell carcinoma under oxidative stress *via* miR-429. Biomed. and Pharmacother. 120, 109460. 10.1016/j.biopha.2019.109460 31550675

[B35] Singh K. Sharma A. Mir M. C. Drazba J. A. Heston W. D. Magi-Galluzzi C. (2014). Autophagic flux determines cell death and survival in response to Apo2L/TRAIL (dulanermin). Mol. Cancer 13 (1), 70. 10.1186/1476-4598-13-70 24655592 PMC3998041

[B36] Singh S. Sharma B. Kanwar S. S. Kumar A. (2016). Lead phytochemicals for anticancer drug development. Front. Plant Sci. 7, 1667. 10.3389/fpls.2016.01667 27877185 PMC5099879

[B37] Sorice M. (2022). Crosstalk of autophagy and apoptosis. Cells 11 (9), 1479. 10.3390/cells11091479 35563785 PMC9102887

[B38] Sung H. Ferlay J. Siegel R. L. Laversanne M. Soerjomataram I. Jemal A. (2021). Global cancer statistics 2020: GLOBOCAN estimates of incidence and mortality worldwide for 36 cancers in 185 countries. CA A Cancer J. Clin. 71 (3), 209–249. 10.3322/caac.21660 33538338

[B39] Tait S. W. G. Green D. R. (2010). Mitochondria and cell death: outer membrane permeabilization and beyond. Nat. Rev. Mol. Cell Biol. 11 (9), 621–632. 10.1038/nrm2952 20683470

[B40] Tong C. Wu Y. Zhang L. Yu Y. (2022). Insulin resistance, autophagy and apoptosis in patients with polycystic ovary syndrome: association with PI3K signaling pathway. Front. Endocrinol. 13, 1091147. 10.3389/fendo.2022.1091147 36589825 PMC9800521

[B41] Tsai C. Y. Kapoor M. Huang Y. P. Lin H. H. Liang Y. C. Lin Y. L. (2016). Synthesis and evaluation of aminothiazole-paeonol derivatives as potential anticancer agents. Molecules 21 (2), 145. 10.3390/molecules21020145 26821004 PMC6273194

[B42] Wu Y. Wang Y. Liu H. Hu Q. Xie Y. Nan X. (2024). Mechanism of apoptosis in oral squamous cell carcinoma promoted by cardamonin through PI3K/AKT signaling pathway. Sci. Rep. 14 (1), 20802. 10.1038/s41598-024-71817-1 39242879 PMC11379709

[B43] Yan M. Wang Q. Yang H. Liu D. Liang W. Chen H. (2025). The paeonol of total glucosides of white Peony regulates the differentiation of CD4+Treg cells through the EP300/Foxp3 axis to relieve pulmonary fibrosis in mice. Cell Biochem. Biophys. 83, 3959–3970. 10.1007/s12013-025-01770-x 40355775

[B44] Yang K. Yue B. Tian H. Wang L. Yang X. Zhang W. (2025). Paeonol inhibits the Glycolysis in oral squamous cell carcinoma though suppressing NAT10-mediated ac4C modification. BMC Cancer 25 (1), 629. 10.1186/s12885-025-14000-7 40197308 PMC11977892

[B45] Ying L. Chen R. Guo R. Liang Y. Hao M. Chen X. (2025). Paeonol suppresses bladder cancer progression *via* apoptotic pathways: insights from *in vitro* and *in vivo* studies. Pharmaceuticals 18 (4), 472. 10.3390/ph18040472 40283909 PMC12030738

[B46] Yonekawa T. Thorburn A. (2013). Autophagy and cell death. Essays Biochem. 55, 105–117. 10.1042/bse0550105 24070475 PMC3894632

[B47] Zhang L. Chen W. X. Li L. L. Cao Y. Z. Geng Y. D. Feng X. J. (2020). Paeonol suppresses proliferation and motility of non-small-cell lung cancer cells by disrupting STAT3/NF-κB signaling. Front. Pharmacol. 11, 572616. 10.3389/fphar.2020.572616 33442382 PMC7797776

[B48] Zhang X. Xu J. Wang X. Xu L. Wang Y. (2025). PI3K-dependent GAB1/Erk phosphorylation renders head and neck squamous cell carcinoma sensitive to PI3Kα inhibitors. Cell Death Dis. 16 (1), 457. 10.1038/s41419-025-07767-x 40533463 PMC12177050

[B49] Zhou C. Zhong W. Zhou J. Sheng F. Fang Z. Wei Y. (2012). Monitoring autophagic flux by an improved tandem fluorescent-tagged LC3 (mTagRFP-mWasabi-LC3) reveals that high-dose rapamycin impairs autophagic flux in cancer cells. Autophagy 8 (8), 1215–1226. 10.4161/auto.20284 22647982

