# Supplementary material for: Paeonol inhibits the development of oral squamous cell carcinoma through the PI3K/AKT signaling pathway
Source: Front Cell Dev Biol. 2026 Mar 4;14:1747424. doi: 10.3389/fcell.2026.1747424 (PMC12996138; doi:10.3389/fcell.2026.1747424)
Supplement: Supplementary file 1 [file DataSheet1.pdf]

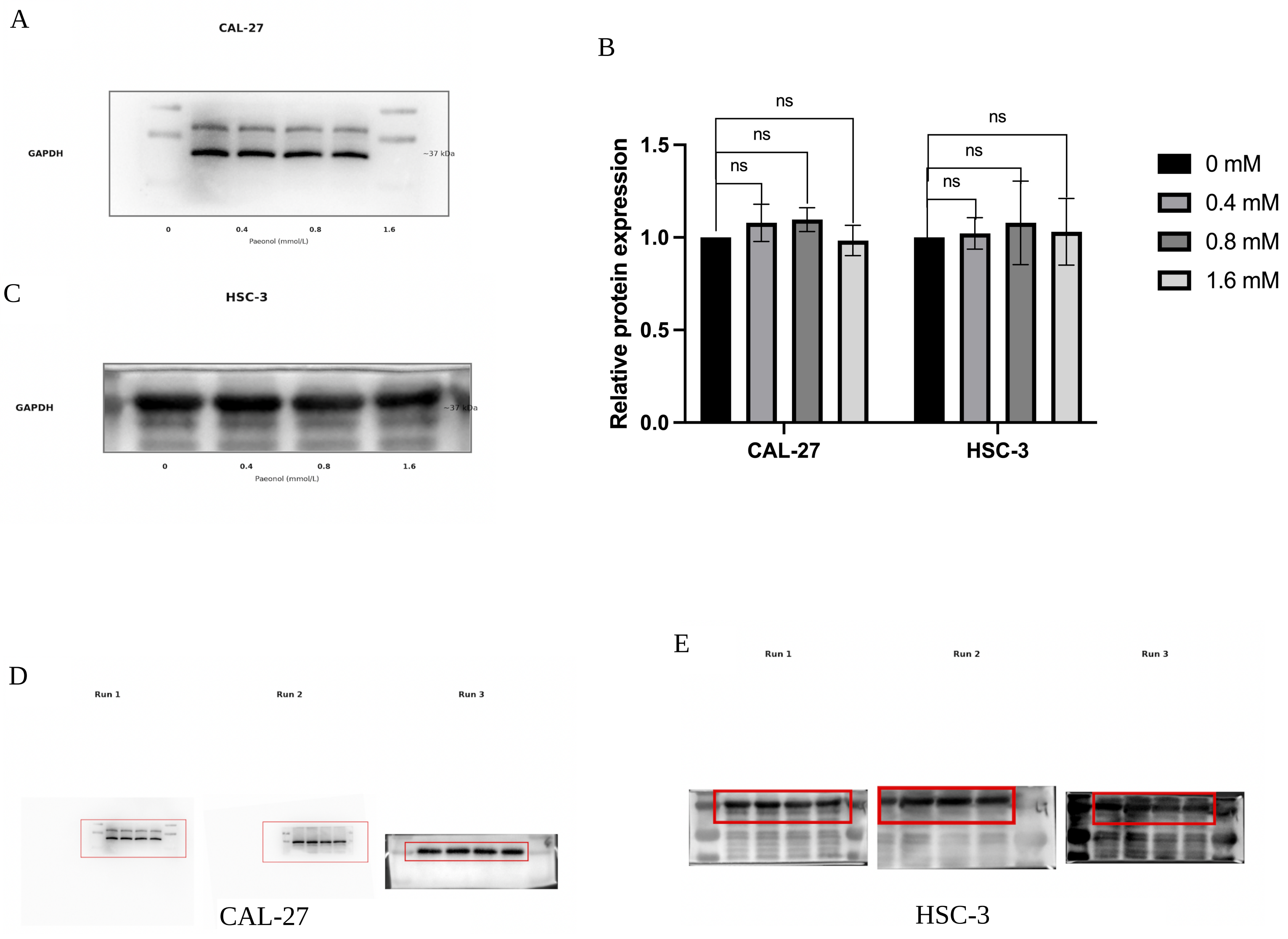

Supplementary Figure S1. GAPDH loading control blots in OSCC cells treated with paeonol. (A、D) CAL-27 cells. (C、E) HSC-3 cells. Cells were treated with paeonol (0, 0.4, 0.8, and 1.6 mmol/L). GAPDH was used as the loading control. Uncropped blots are provided with red boxes indicating the regions shown in panels A and C. Densitometric values (panels B) are shown for three independent immunoblot runs and were normalized to the 0 mmol/L lane within each run. No inferential statistics were applied.
